# A Comparative Study of Case-Based Learning (CBL) Versus the Traditional Teaching Method for Enhanced Analysis and Interpretation of Electrocardiogram (ECG) Among Medical Students

**DOI:** 10.7759/cureus.79587

**Published:** 2025-02-24

**Authors:** Sanket Makwana, Hemal Sarvaiya, Vimalkumar Dalsaniya

**Affiliations:** 1 General Medicine, C. U. Shah Medical College, Surendranagar, IND

**Keywords:** case-based learning (cbl), electrocardiogram (ecg), rhythm abnormalities, traditional didactic learning (tdl), waveform abnormalities

## Abstract

Background and objective

Learning electrocardiogram (ECG) analysis and interpretation is essential for medical students, but traditional didactic learning (TDL) methods often fall short. This study explores case-based learning (CBL), which immerses students in real-world scenarios and compares its effectiveness to traditional methods for enhancing ECG skills among medical students.

Materials and methods

This prospective educational interventional study included the phase III part II Bachelor of Medicine and Bachelor of Surgery (MBBS) students conducted from February 2024 to October 2024 at C. U. Shah Medical College, Surendranagar, India. After obtaining ethical approval, students were divided into two equal groups using simple random sampling. In the first session, Group A was taught ECG rhythm abnormalities via CBL, while Group B was taught via TDL. In the second session, Group A was taught ECG waveform abnormalities via TDL, while Group B was taught CBL. Pre- and post-assessment multiple-choice question (MCQ) tests were conducted, along with student and faculty feedback collected using a five-point Likert scale to compare CBL and TDL.

Results

Statistical analysis shows higher immediate post-test scores with CBL than TDL, supported by highly significant p-values. The students who have taught with CBL have retained significantly higher ECG competence, supported by higher mean scores in delayed assessment tests than those who taught TDL. Both students and teachers perceive CBL positively, indicating it enhances academic performance and is well-received.

Conclusion

CBL is significantly more effective than TDL in improving the understanding of rhythm and waveform ECG abnormalities. Incorporating CBL into medical education is recommended to enhance learning outcomes, especially in complex areas like rhythm and waveform ECG abnormalities.

## Introduction

An electrocardiogram (ECG) is an essential tool for cardiovascular screening in emergency medical departments. Thus, ECG analysis and interpretation need to play a vital role in medical professionals' diagnosis and management of cardiac diseases [1,2]. ECG teaching and interpretation has been defined as essential competency in competency-based medical education (CBME) for Indian medical graduates (IMG). If medical students do not diagnose ECG correctly, it will hamper their clinical diagnosis and lead to poor patient outcomes. However, the major problem in our medical education system is that most medical students do not have sufficient knowledge or confidence in analysing and interpreting ECG [3-5]. 

In the past, our medical students have typically relied on traditional didactic learning (TDL) methods such as classroom-based lectures and textbook-based teaching, focusing on concepts behind ECGs with minimal time allotted to students for actual interpretation of ECGs [6]. In clinical rotations, students usually see the actual patients and ECGs. Still, this exposure differs for every student as they will only see a random sample of patients in their rotations, so they might not get exposure to various ECGs in their clinical tenure [7]. Hence, it is a time to revisit the conventional methods of ECG teaching to medical students and implement more student-friendly and quickly applicable methods for enhanced ECG analysis and interpretation of ECG among medical students [3]. 

Case-based learning (CBL) is a novel method that explores real-life scenarios using the case as a trigger to promote a higher level of cognition [8,9]. CBL provides students with detailed patient history and physical examination findings with relevant laboratory and radiological data that enhance their decision-making [10]. Thus, CBL can improve critical thinking and problem-solving approaches and will merge their theoretical knowledge with actual clinical application in real-world scenarios [9,11]. Students will gain more interest and confidence with such methods as they find them more engaging and satisfactory, as they feel like they are dealing with clinical cases rather than classroom-based didactic learning [12,13]. 

There are numerous methods worldwide to teach ECG and improve ECG interpretation skills, including Internet-based e-learning methods. ECG teaching is uniquely challenging as no established standard method is acknowledged worldwide as evidence-based and the most effective method in medical education [14,15]. Considering the challenge of ECG interpretation, we have conducted this study to compare CBL methods versus traditional methods for enhanced ECG analysis and interpretation among medical students. 

## Materials and methods

Study design

This is a prospective educational interventional study that compares CBL with traditional teaching methods for enhanced analysis and interpretation of ECG among medical students. 

Study participants

Phase III part II MBBS students were enrolled in the study after written informed consent.

Study duration and study site

This study spanned from February 2024 to October 2024 at C. U. Shah Medical College and Hospital, Surendranagar, Gujarat, India. 

Intervention

Before starting the study, we obtained institutional ethical permission from the Institutional Ethics Committee (reference no. CUSMC/IEC(HR)/Pro.Approval-RP-06/2024/OUT-18/2024). Students and faculty were sensitized before the study commenced. After obtaining written informed consent, all the students were randomly assigned into two equal groups (Group A and Group B) using a simple random sampling method. A computer-generated randomization sequence was used to ensure unbiased allocation.

In the first session, Group A was taught ECG rhythm abnormalities via CBL in further subdivided groups according to the standard lesson plan prepared. By contrast, Group B was taught the same topic via a traditional didactic interactive lecture. In the second session, the teaching methods were switched: Group A was taught ECG waveform abnormalities via a traditional interactive lecture, while Group B received the same topic via CBL.

This crossover design ensured all students were exposed to both teaching methods, minimising inter-group variability. The instructors for both groups were experienced faculty members, ensuring consistency in content delivery and teaching quality.

Study tool 

Pre- and post-assessment multiple-choice questions (MCQs) were utilized as a study tool. MCQs have included questions related to basic knowledge of ECG, its application, and real-time patient ECG strips for case-based MCQs [16]. Each test comprised 10 single-best-answer MCQs. Each correct question was awarded one mark, and a negative mark did not apply. These MCQs underwent pre-validation for their contents, time requirement, clarity of instructions, and appropriateness by senior faculty members of our department and the Medical Education Unit (MEU) team. For feedback and perception, the five-point Likert scale was utilized (see Appendix). 

Data collection

During the study, the participants were asked to complete three assessment tests. The first MCQ test was a pre-intervention test to determine baseline (pre-existing) ECG competence. The second MCQ test was an immediate post-intervention test to assess the participants' acquisition of ECG competence. Pre- and post-intervention tests were conducted for both topics (rhythm abnormalities and waveform abnormalities) in both groups during their respective session. The third MCQ test was a delayed post-intervention test performed after six months for both groups for their respective topics to decide their retention of knowledge about ECG interpretation. In the end, student and faculty feedback was taken using the five-point Likert scale for CBL over the traditional teaching method. 

Statistical analysis

We utilized suitable statistical tests to compare the performance outcomes of traditional and CBL methods. Parametric data were summarised as means with standard deviations (SD), while a median with interquartile range (IQR) was used for non-parametric data. Paired and unpaired t-tests assessed within-group and between-group differences in test scores, respectively. Categorical variables were expressed as frequencies and percentages. After data collection, statistical analysis was performed using IBM SPSS Statistics for Windows, Version 28.0 (released 2021, IBM Corp., Armonk, NY). A p-value of <0.05 was considered statistically significant.

## Results

This educational interventional study included the participants from phase III part II MBBS students at C. U. Shah Medical College Surendranagar. It included two sessions: one on ECG rhythm abnormalities and the second on waveform abnormalities. 

The first session was conducted for ECG rhythm abnormalities. The 84 students enrolled for the first session were equally divided into Group A and Group B. Group A was taught ECG using the CBL method, and Group B was taught ECG using TDL. The mean pre-test score for Group A (CBL), 2.572, was increased to 8.309 at the immediate post-intervention test. For Group B, the mean score on the pre-test was 2.286, which rose to 6.952 on the immediate post-test. The mean post-test score was higher in Group A (CBL) than in Group B (TDL). The p-value for within-the-group comparison is highly significant in both groups (Table 1).

**Table 1:** Comparison of pre-test and immediate post-test scores within the group for rhythm abnormalities SD: standard deviation; n: number. Paired t-test used for within-group comparison; p-value <0.05 considered statistically significant.

Study group	Pre-test		Immediate post-test		p-value
	Mean	SD	Mean	SD	
Group A (CBL) n = 42	2.571	±1.150	8.309	±1.137	<0.00001
Group B (TDL) n = 42	2.286	±1.088	6.952	±1.147	<0.00001

ECG waveform abnormalities were taught in the second session. A total of 88 students attended the second session, subdivided equally into Group A and Group B. Group A was taught by TDL, while Group B was taught by the CBL method for ECG. The mean pre-test score for Group A was 2.795, while for Group B, it was 3.025. The immediate post-intervention score for Group B (CBL) was 9.023, compared to 7.727 in Group A (TDL). The p-value was statistically significant for within-group comparison (p < 0.001) (Table 2). 

**Table 2:** Comparison of pre-test and immediate post-test scores within the group for waveform abnormalities SD: standard deviation; n: number. Paired t-test used for within-group comparison; p-value < 0.05 considered statistically significant.

Study group	Pre-test		Post-test		p-value
	Mean	SD	Mean	SD	
Group A (TDL) n = 44	2.795	±1.002	7.727	±1.167	<0.00001
Group B (CBL) n = 44	3.023	±1.023	9.023	±0.849	<0.00001

The immediate post-test scores between the two groups, rhythm and waveform abnormalities, were compared using an unpaired t-test. Among rhythm abnormalities, the mean score for Group A (CBL) was 8.309, higher than 6.952 in Group B (CBL). In the waveform abnormalities session, the mean score of Group B (CBL) was 9.023, significantly higher than Group A (TDL)'s mean score of 7.727. The p-value was statistically significant between the group comparisons in the post-intervention test (Table 3). The effect size observed in the immediate post-test result for rhythm and waveform abnormalities has a large effect size (Cohen's d = 1.188 and 1.270, respectively), indicating a strong impact of CBL on immediate learning outcomes.

**Table 3:** Comparison of immediate post-test scores between the two groups for rhythm and waveform abnormalities SD: standard deviation; n: number. Unpaired t-test used for between the group comparison; p-value < 0.05 considered statistically significant.

Data	Rhythm abnormalities		Waveform abnormalities	
	Immediate post-test		Immediate post-test	
	Group A (CBL)	Group B (TDL)	Group A (TDL)	Group B (CBL)
Mean	8.309	6.952	7.727	9.023
SD	±1.137	±1.147	±1.167	±0.849
t-statistics	5.4474		-5.9494	
Significance (p-value)	<0.00001		<0.00001	

The delayed post-intervention test was conducted to test knowledge retention regarding ECG analysis and interpretation skills. Here, we found the mean score for Group A (CBL) in rhythm abnormalities was 6.69, somewhat higher than 5.801 in Group B (TDL). The p-value for rhythm abnormalities was 0.03886. In waveform abnormalities, the mean score for Group B (CBL) was 8.227, significantly higher compared to 6.841 in Group (TDL) (Table 4). At six months, the CBL group has observed moderate to large effect sizes (Cohen's d = 0.601 for rhythm and Cohen's d = 1.147 for waveform abnormalities), leading to superior long-term retention, particularly for waveform abnormalities.

**Table 4:** Comparison of delayed post-test scores at six months between the two groups for rhythm and waveform abnormalities SD: standard deviation; n: number. Unpaired t-test used for between the group comparison; p-value < 0.05 considered statistically significant.

Data	Rhythm abnormalities		Waveform abnormalities	
	Post-test (six months)		Post-test (six months)	
	Group A (CBL)	Group B (TDL)	Group A (TDL)	Group B (CBL)
Mean	6.69	5.801	6.841	8.227
SD	±1.239	±1.685	±1.256	±1.159
t-statistics	2.72894		-5.38046	
Significance (p-value)	0.03886		<0.00001	

**Figure 1:**
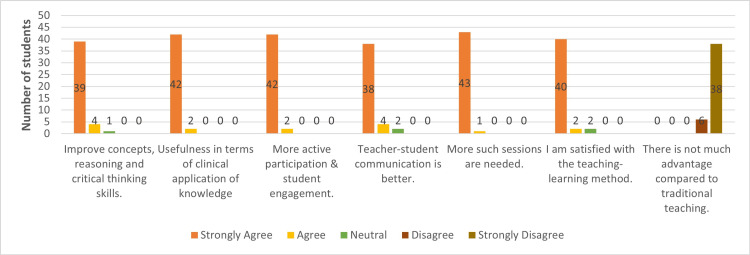
Perceptions of students regarding case-based learning over traditional teaching (n = 44) n: number

For feedback and perception, the five-point Likert scale was utilized. Here, the highest score was 5 for strongly agreeing, and the lowest score was 1 for strongly disagreeing regarding their perception of CBL over traditional teaching methods for ECG. Most students felt that CBL is more beneficial in improving concepts, reasoning skills, critical thinking, and clinical application. They favored CBL as more engaging for students, enhancing communication with teachers, and demanded more sessions with CBL (Figure 1). 

**Figure 2:**
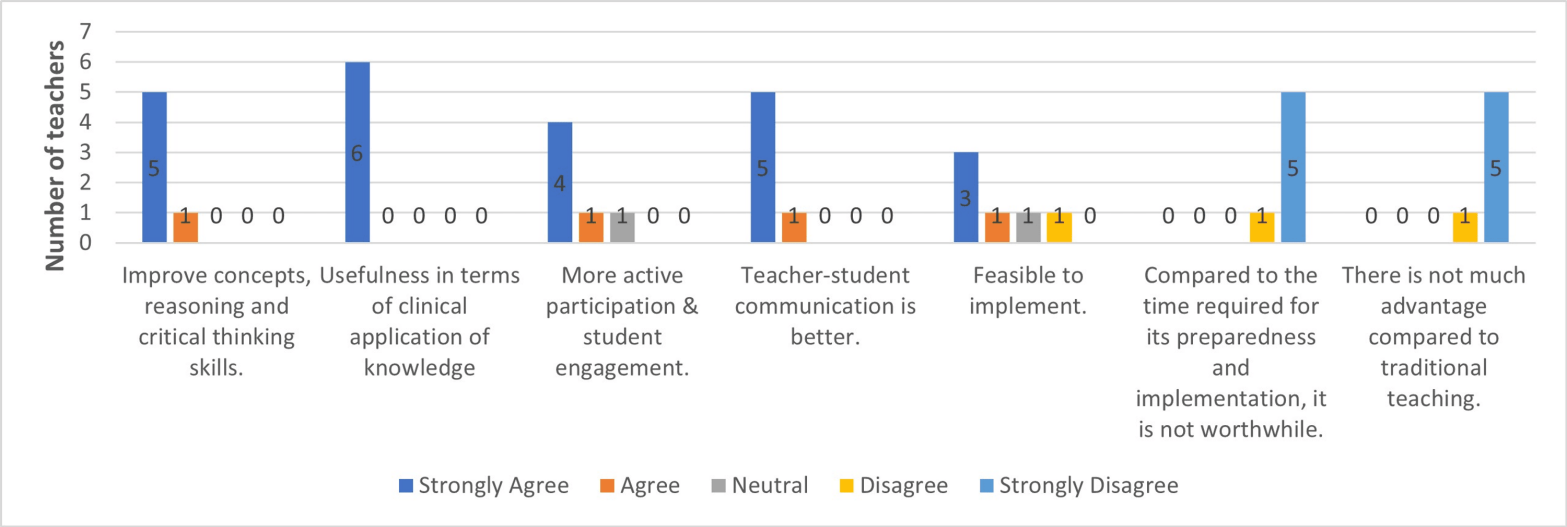
Perceptions of teachers regarding case-based learning over traditional teaching (n = 6) n: number

We have also used the five-point Likert scale to assess teachers' feedback and perceptions of CBL compared to traditional teaching methods. Most teachers agree with students that CBL improves students' concepts, critical thinking, reasoning skills, and application in clinical scenarios. Most teachers also suggested that CBL enhances communication skills and is advantageous. Still, it requires much more time to prepare and implement CBL methods than traditional teaching methods (Figure 2). 

## Discussion

This educational interventional study aimed to compare CBL with TDL to improve students' ability to analyze ECG and interpret it correctly. Traditional teaching methods, such as classroom-based or interactive didactic lectures, remain the predominant approach for ECG instruction [6]. However, it often fails to enhance the student's ECG competence and active engagement in diagnosing various clinical cardiac conditions based on correct ECG interpretation [17,18]. Students often fail to apply the theoretical knowledge of ECG teaching during their clinical application. This educational gap must be addressed so students can master their ECG skills and have expertise in correctly interpreting the ECG [17,18].

Various methods are available for ECG teaching in medical education, but no single approach is universally recognized as the most effective [14]. Studies have explored multiple strategies, including blended learning (a combination of online modules and interactive case discussions), case-based e-learning, and simulation-based training [3,19]. These findings highlight the need for interactive and student-centered teaching approaches to optimize ECG education.

CBL is an educational teaching method that incorporates real-life scenarios to improve students' academic excellence by helping them merge their theoretical knowledge with clinical application and cultivating their reasoning and critical thinking skills [20]. The CBL method also improves students' engagement in learning through better communication between teachers and learners and makes the process more interesting [21]. Hence, we tried implementing CBL for enhanced ECG analysis and interpretation among medical students.

Our study observed that the CBL and TDL methods significantly improve students' post-assessment scores during the immediate post-test. However, the immediate mean post-assessment score is substantially higher in CBL than in TDL for both the session of rhythm and waveform ECG abnormalities. This result is statistically highly significant. This study's results are consistent with those obtained by Elkammash et al., which show the superiority of CBL over traditional lectures for ECG interpretation skills [19]. Another study by Wen et al. also concluded that the CBL method is very innovative and effective, improving students' capabilities for ECG analysis and interpretation [22]. Our study demonstrates that CBL is significantly more effective than TDL in improving ECG interpretation skills, with large effect sizes observed in immediate post-test results (Cohen's d = 1.188 for rhythm abnormalities and d =1.270 for waveform abnormalities. This suggests that CBL not only enhances short-term learning but does so with a substantial magnitude.

Our study assessed the retention of knowledge about ECG competence by taking the assessment test at six months. The literature indicates that competence tends to decrease over time without regular teaching and review of the topic [23,24]. We have seen similar findings in our study. We have not exposed the students to ECG teaching between immediate and delayed post-tests at six months; we have observed a decrease in mean post-test scores in both sessions with both teaching methods. However, the students who have taught with CBL have retained significantly higher competence than those who taught TDL. This observed value is statistically highly significant. This suggests that the CBL method can improve their ECG analysis and interpretation skills immediately and retain their knowledge for longer durations. In their study, Viljoen et al. also observed equivalent results on retaining knowledge about ECG competence at six months [3]. Additionally, knowledge retention at six months remained higher in the CBL group with moderate-to-large effect sizes (Cohen's d = 0.601 for rhythm and d = 1.147 for waveform abnormalities). These findings with large effect sizes further reinforce the practical significance of integrating CBL into ECG education for medical students.

In our study, the perception and feedback regarding CBL over TDL are more favorable toward CBL. Most students found the CBL method more helpful in improving their clinical concepts, critical thinking, and reasoning skills. They considered the CBL method more like the clinical application of their knowledge in real patient scenarios. CBL improved their communication with teachers and asked for more such sessions. Teachers' perceptions and feedback also coincide with students' feedback. However, most teachers commented that this CBL method requires more time and expertise than didactic lectures, although they agreed that this CBL should be used more for such clinical teaching topics. A study by Garvey et al. also described that most students agreed that CBL improves student-doctor communication and problem-solving skills compared to traditional teaching methods [25]. Our findings are also consistent with the similar perception feedback obtained by Nishal et al. in the comparative study of CBL vs. traditional teaching methods [26].

Our study has several limitations that can be addressed in future research. We have a smaller sample size (n = 44 students, n = 6 teachers). This smaller sample size limits its generalizability to large populations. It increases error risk, and the study requires a considerable sample size and a diverse sample that can improve its confidence level. Another limitation is the crossover design, which, minimizing inter-group variability, may have introduced learning carryover effects. In addition, it restricts the ability to assess each method's long-term impact independently. Future studies should use a parallel-group design with larger multicenter cohorts to provide a more precise comparison of CBL over TDL.

## Conclusions

CBL significantly enhances ECG interpretation skills compared to TDL, as demonstrated by higher immediate post-test scores for rhythm and waveform ECG abnormalities. Knowledge retention at six months was also superior with CBL for rhythm and waveform abnormalities. This is supported by highly significant p-values (p < 0.00001) and strong effects on immediate learning and long-term retention. Student and faculty feedback strongly favored CBL's effectiveness in improving critical thinking and clinical application. Given these statistically significant benefits, incorporating CBL into medical education is recommended to enhance learning outcomes, especially in complex areas like rhythm and waveform ECG abnormalities.

## Appendices

Five-point Likert scale for case-based learning over the traditional teaching method 

**Table 5:** Perceptions of students regarding case-based learning over traditional teaching Five-point Likert scale: (strongly agree = 5, agree = 4, neutral = 3, disagree = 2, strongly disagree = 1)

	Strongly agree	Agree	Neutral	Disagree	Strongly disagree
Improve concepts, reasoning, and critical thinking skills					
Usefulness in terms of clinical application of knowledge					
More active participation and student engagement					
Teacher-student communication is better					
More such sessions are needed.					
I am satisfied with the teaching-learning method.					
There is not much advantage compared to traditional teaching.					

**Table 6:** Perceptions of teachers regarding case-based learning over traditional teaching Five-point Likert scale: (strongly agree = 5, agree = 4, neutral = 3, disagree = 2, strongly disagree = 1)

	Strongly agree	Agree	Neutral	Disagree	Strongly disagree
Improve concepts, reasoning, and critical thinking skills					
Usefulness in terms of clinical application of knowledge					
More active participation and student engagement					
Teacher-student communication is better.					
Feasible to implement.					
Compared to the time required for its preparedness and implementation, it is not worthwhile.					
There is not much advantage compared to traditional teaching.					
